# Outcome Disparities Between Medical Personnel and Nonmedical Personnel Patients Receiving Definitive Surgery for Colorectal Cancer: A Nationwide Population-Based Study

**DOI:** 10.1097/MD.0000000000000402

**Published:** 2015-01-30

**Authors:** Chia-Jen Liu, Nicole Huang, Chun-Chi Lin, Yu-Ting Lee, Yu-Wen Hu, Chiu-Mei Yeh, Tzeng-Ji Chen, Yiing-Jenq Chou

**Affiliations:** From the Division of Hematology and Oncology (C-JL, Y-TL), Department of Medicine, Taipei Veterans General Hospital; School of Medicine (C-JL, C-CL, Y-WH, T-JC); Institute of Public Health (C-JL, Y-JC); Institute of Hospital and Health Care Administration (NH), National Yang-Ming University; Division of Colon and Rectal Surgery (C-CL), Department of Surgery; Cancer Center (Y-WH); Department of Family Medicine (C-MY, T-JC), Taipei Veterans General Hospital, Taipei, Taiwan.

## Abstract

Disparities in quality of care have always been a major challenge in health care. Providing information to patients may help to narrow such disparities. However, the relationship between level of patient information and outcomes remains to be explored. More importantly, would better-informed patients have better outcomes through their choice of higher quality providers? We hypothesize that medical professionals may have better outcomes than nonmedical professionals following definitive surgery for colorectal cancer (CRC), and their choice of provider may mediate this relationship.

We identified 61,728 patients with CRC receiving definitive surgery between 2005 and 2011 from the Taiwan National Health Insurance Research Database. Medical professionals were identified via the registry for medical personnel. Indicators for surgical outcome such as emergency room (ER) visits within 30 days, medical expenses, length of hospital stay (LOS), and 5-year mortality were analyzed by using fixed and random effects multivariate regression models.

Compared with nonmedical personnel CRC patients, a greater proportion of medical personnel received definitive surgery from higher volume surgeons (median 390 vs 311 within the study period) and/or in higher volume hospitals (median 1527 vs 1312 within the study period). CRC patients who are medical personnel had a shorter median LOS (12 vs 14 days), lower median medical expenses (112,687 vs 121,332 New Taiwan dollars), a lower ER visit rate within 30 days (11.3% vs 13.0%), and lower 5-year mortality. After adjusting for patient characteristics, medical personnel had a significantly lower hazard of 5-year mortality, and were significantly more likely to have a LOS shorter than 14 days than their nonmedical personnel counterparts. However, after adjusting for patient and provider characteristics, while medical personnel were significantly less likely to have a long LOS, no significant difference was observed in 5-year mortality between the 2 groups.

Medical personnel did have a significantly better survival outcome and a shorter length of stay following definitive surgery than nonmedical personnel patients. The outcome disparities can be partially explained by characteristics of their treatment providers. The findings may serve as an important reference for better understanding how information may narrow gaps in quality of care through better choice of providers.

## INTRODUCTION

Colorectal cancer (CRC) is a global issue and the highest incident cancer in Taiwan. Effectiveness of screening for CRC reduces mortality through early detection of CRC and multidisciplinary treatment.^1^ Definitive surgery is the backbone of multidisciplinary CRC treatment. The 5-year survival of patients with CRC receiving definitive surgery has seen remarkable improvement.^2^

Colectomy is a common definitive surgery and can be performed in different levels of hospitals. The circumferential resection margin is a prognostic factor for both local recurrence and overall survival.^3^ In addition, a large prospective trial and 2 population-based studies proved that an increase in the number of lymph nodes evaluated can improve survival through accurate staging and tailored treatments.^4–6^ An increasing body of research has consistently documented that surgical complications and long-term survival rate are correlated to providers’ volume.^7–9^ Surgery-related deaths can be avoided if cancer surgeries are performed by high-volume providers.^10^ In addition to the “practice-makes-perfect” hypothesis, high-volume providers also often adopt multidisciplinary approaches,^11^ adhere to guidelines,^12^ and provide high treatment quality.^13^

Medical personnel, presumably the better informed consumers of health care, might have different care-seeking behaviors and utilization patterns of health care service.^14^ Possibly due to either limited provider performance information being available to the public or multiple difficulties in the public's understanding or ability to translate the performance information, many may seek advice from their medical professional friends or relatives when choosing their surgical providers.^15^ Medical personnel are believed to be the “insiders” of medical care systems, and have better understanding or information about reputations and the outcomes of providers. However, it is unclear whether medical personnel do have better outcomes following definitive surgeries for colorectal cancer (CRC). In addition, if they do, are the better outcomes mediated through their choices of surgical providers?

The National Health Insurance (NHI) program in Taiwan is a single-payer, mandatory universal social insurance, which provides comprehensive coverage to all Taiwanese residents. Its comprehensive coverage of cancer care with no or very limited cost-sharing substantially reduces financial burdens of the patients with CRC. In addition, the claims data collected by the NHI program allow for comparing possible outcome disparities between general patients and patients who were medical personnel following definitive surgeries for CRC, and investigating whether the differences in choice of surgical providers contribute to the outcome disparities.

## PATIENTS AND METHODS

### Data Sources

Taiwan's NHI program was established in 1995, and its coverage includes outpatient, inpatient, emergency, dental, and traditional Chinese medical services, as well as prescription medicine. The National Health Insurance Research Database (NHIRD) provides nationwide data for health research. The registry for catastrophic illness patients (RCIP) integrates multiple NHI databases, including NHI enrolment files, claims data, and the registry for drug prescriptions, to provide comprehensive information on NHI enrolment and utilization of health care resources of all patients with severe diseases, of which cancer is included. Patients with catastrophic illness receive copayment exemption under the NHI program.

All NHIRD information that can potentially identify an individual patient is encrypted. All data is confidential, as required by the Bureau of National Health Insurance and the National Health Research Institute policies. This study was approved by the Institutional Review Board of the Taipei Veterans General Hospital, Taipei, Taiwan (No. 2014-05-002BE).

### Study Population

**Figure 1:**
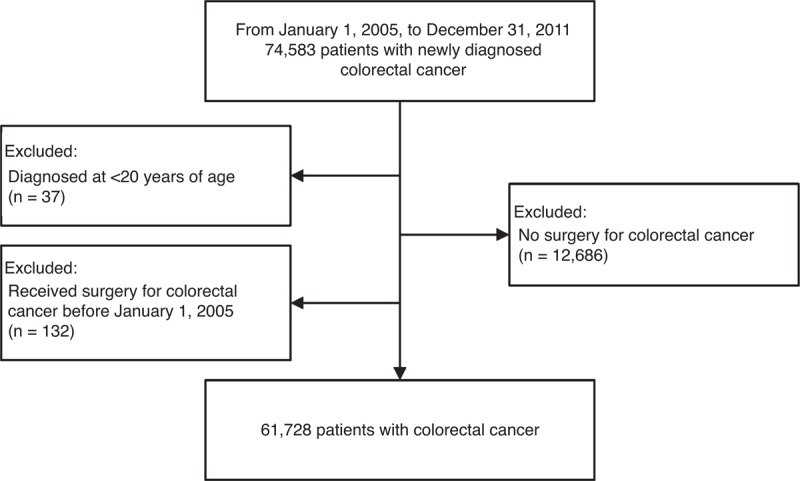
Patient selection flow chart.

Patients with newly diagnosed CRC were retrieved via the RCIP according to the International Classification of Diseases, Ninth Revision, Clinical Modification (ICD-9-CM) major codes 153 and 154 from January 1, 2005, to December 31, 2011. Diagnosis of CRC requires pathological confirmation for enrolment in the RCIP. Those diagnosed with CRC before January 1, 2005, were not enrolled. Patients with CRC diagnosed at <20 years old and those without definitive surgery during the study period were excluded (Figure 1). Definitive surgery for CRC means colectomy with or without metastasectomy.

### Variables

The main dependent variable was occurrence of death within 5 years since the first date of definitive surgery, which is a common indicator for the long-term outcome of cancer surgery.^16–20^ The other indicators for the outcome and resource utilization included length of hospital stay (LOS), emergency room (ER) visits within 30 days, and medical costs for definitive surgery and related expenditures (medical expenses). All patients enrolled in this study were followed until death, dropout from the NHI program, or the end of the year 2011.

The medical personnel were identified from the Registry for Medical Personnel. The medical personnel enrolled in the registry are required to have a license for medical occupations, such as doctors, nurses, pharmacists, physical therapists, dietitians, medical technologists, and others. Surgeons’ and hospitals’ volume were calculated according to the claims made by providers for CRC definitive surgery. We calculated the volume of CRC definitive surgeries for each hospital and surgeon. The median volume of CRC definitive surgeries was 1,312 (interquartile range [IQR] 530–2,623) for hospitals and 321 (IQR 180–560) for surgeons. We divided all patients into 3 tertiles according to hospitals’ and surgeons’ volume, respectively. Information on comorbidities, including hypertension, diabetes mellitus (DM), heart failure, chronic obstructive pulmonary disease (COPD), end-stage renal disease (ESRD), dyslipidemia, liver cirrhosis, and cerebral vascular accidents (CVAs), was collected from NHIRD. Socioeconomic status was categorized by degree of urbanization and income.

### Statistical Analyses

Demographic data, including age, sex, comorbidity, degree of urbanization, and income, were compared between CRC patients as medical personnel or not by using Fisher exact test or the χ^2^ test for categorical variables, and the Mann–Whitney U test for continuous variables. Univariate and multivariate Cox proportional hazards models were used to identify predictors of mortality among CRC patients. In a similar method, logistic regression models were used to identify risk factors for hospital stay at least median, and ER visits within 30 days, and linear regression models were performed to estimate medical expenses. All factors with *P* < 0.1 in the univariate analyses were included in the multivariate analysis. The surgeon-level random effects were adjusted by using a frailty model for Cox regression and a generalized estimating equation for linear and logistic regression in the multivariate analysis.^21,22^ Data were extracted and computed using the Perl programming language (version 5.12.2). Microsoft SQL Server 2012 (Microsoft Corp, Redmond, WA) was used for data linkage, processing, and sampling. All statistical analyses were performed using SAS 9.3 software (SAS Institute Inc, Cary, NC). Statistical significance was defined as a *P* value of <0.05.

## RESULTS

### Characteristics of the Study Population

We identified 74,583 patients with CRC in the RCIP from January 1, 2005, to December 31, 2011. Of these, 37 patients were <20 years of age, 12,686 patients never received definitive surgery for CRC, and 132 patients received surgery for CRC before January 1, 2005. Therefore, the final cohort consisted of 61,728 patients, including 34,996 (56.7%) males and 26,732 (43.3%) females, of median age 66 years at diagnosis (IQR 55–75 years). Overall, this cohort was observed for 149,518 person-years from 2005 to 2011. There were 480 patients as medical personnel included in this study. Age, sex, socioeconomic status, and underlying comorbidities were different between medical personnel and nonmedical personnel. Medical personnel had a higher proportion of choosing high-volume hospitals and surgeons. The characteristics of hospitals and surgeons are compared in Table 1.

**Table 1:**
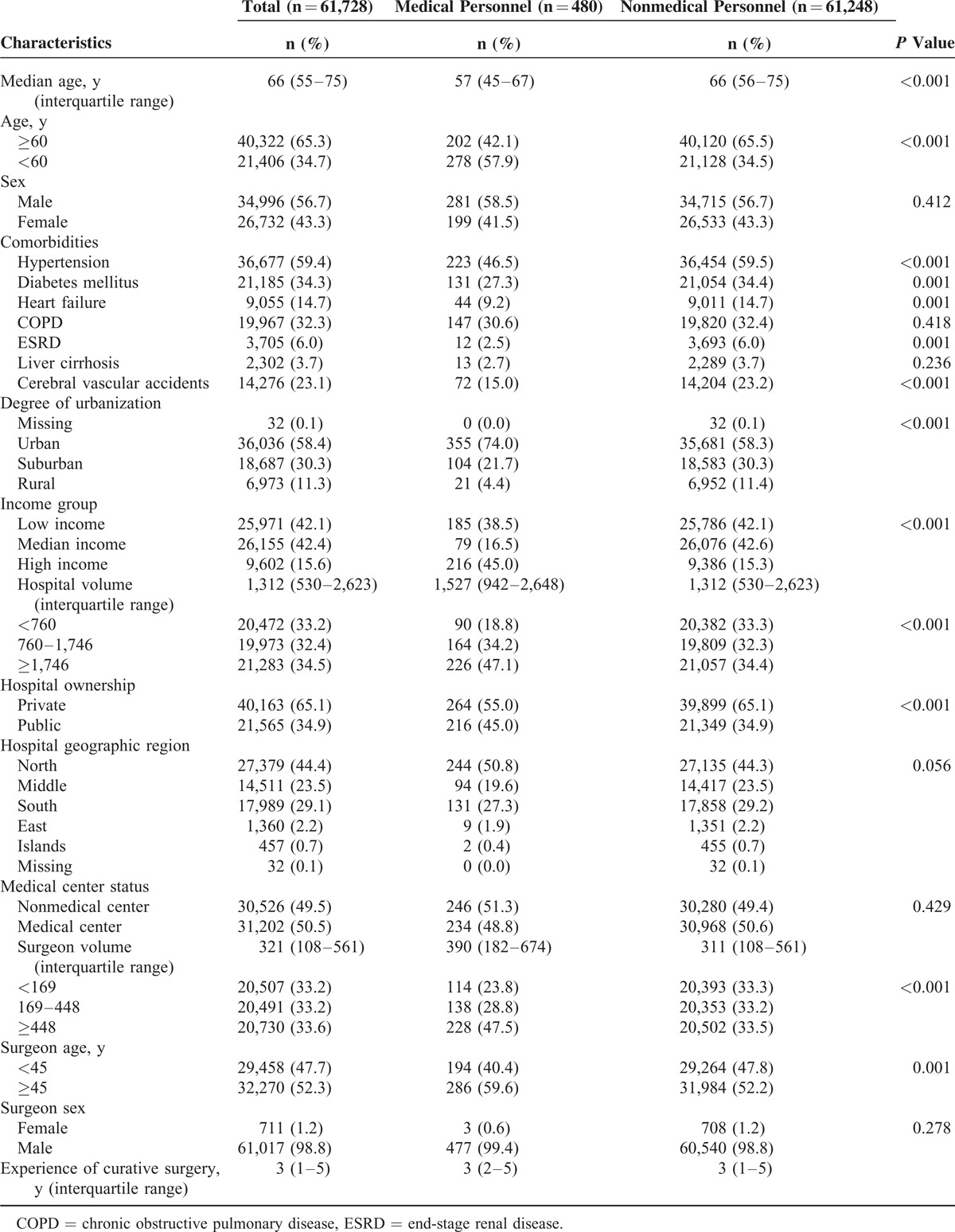
Baseline Characteristics of Patients With Colorectal Cancer

### Differences in ER Visits Within 30 Days, Medical Expenses, and Hospital Stay

After adjusting for individual characteristics, medical personnel patients had a significantly lower risk of having ER visits within 30 days of discharge and lower medical expenses. The differences are illuminated after taking consideration of provider characteristics in Model 2. Furthermore, the median LOS was 14 days. Medical personnel had a significantly lower risk of having a LOS longer than the median LOS following definitive surgery for CRC compared with nonmedical personnel after adjusting for characteristics of patients (adjusted odds ratio [OR] 0.68, 95% confidence interval [CI] 0.56–0.82; *P* < 0.001; Model 1). Similarly, after adjusting for both individual and provider characteristics, medical personnel patients continued to show a significantly lower risk of having a LOS longer than the median LOS following definitive surgery for CRC (adjusted OR 0.74, 95% CI 0.60–0.91; *P* = 0.005; Model 2). The results are summarized in Table 2.

**Table 2:**
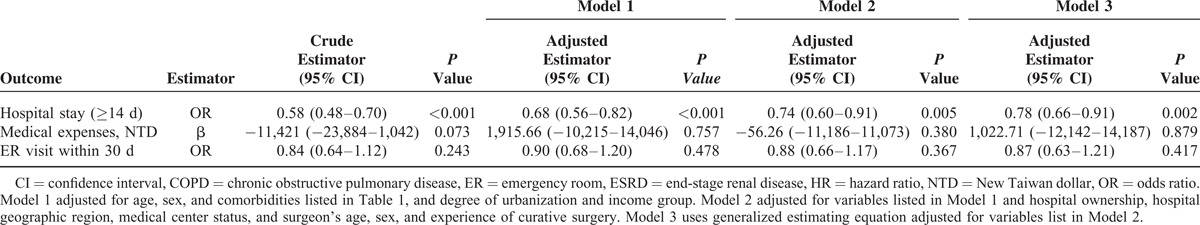
Different Outcome Development Between Medical Personnel and Nonmedical Personnel With Colorectal Cancer

### Differences in 5-Year Mortality

**Figure 2:**
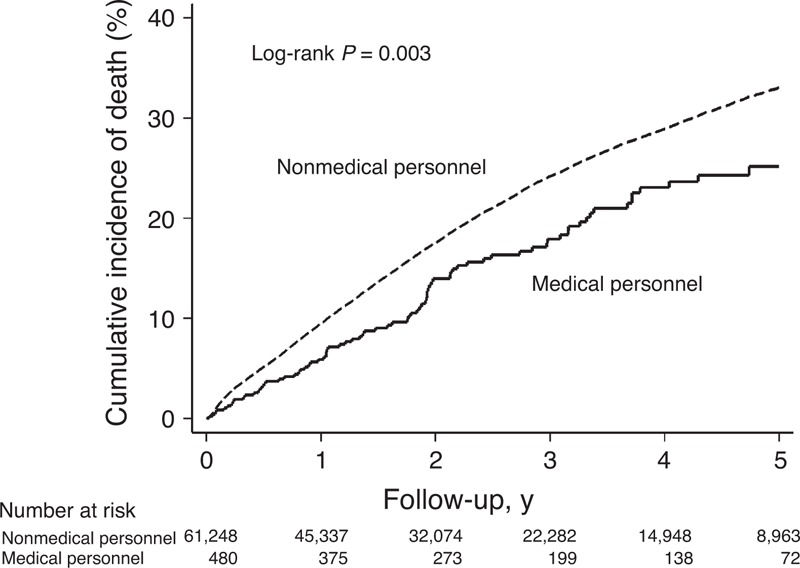
Cumulative incidence of death between medical personnel and nonmedical personnel with colorectal cancer.

Medical personnel with CRC had significantly lower 5-year all-cause mortality than nonmedical personnel did (25.2% vs 33.0%; *P* = 0.003) (Figure 2). The univariate Cox proportional hazards analysis showed that age ≥60 years, being male, having hypertension, DM, liver cirrhosis, heart failure, ESRD, COPD, CVA, lower urbanization, and lower income, as well as being nonmedical personnel were significantly associated with a higher risk of mortality within the 5-year observation period. After adjustment for the individual characteristics mentioned above, being medical personnel still showed a significantly lower hazard of death (adjusted hazard ratio [HR] 0.80, 95% CI 0.64–0.99; *P* = 0.04; Model 1). After taking consideration of both individual and provider characteristics using the frailty model (Model 2), the mortality difference between medical and nonmedical personnel substantially reduced and was no longer statistically significant (adjusted HR 0.96, 95% CI 0.78–1.18; *P* = 0.70). Being medical personnel had no significant difference on death. The details are shown in Table 3.

**Table 3:**
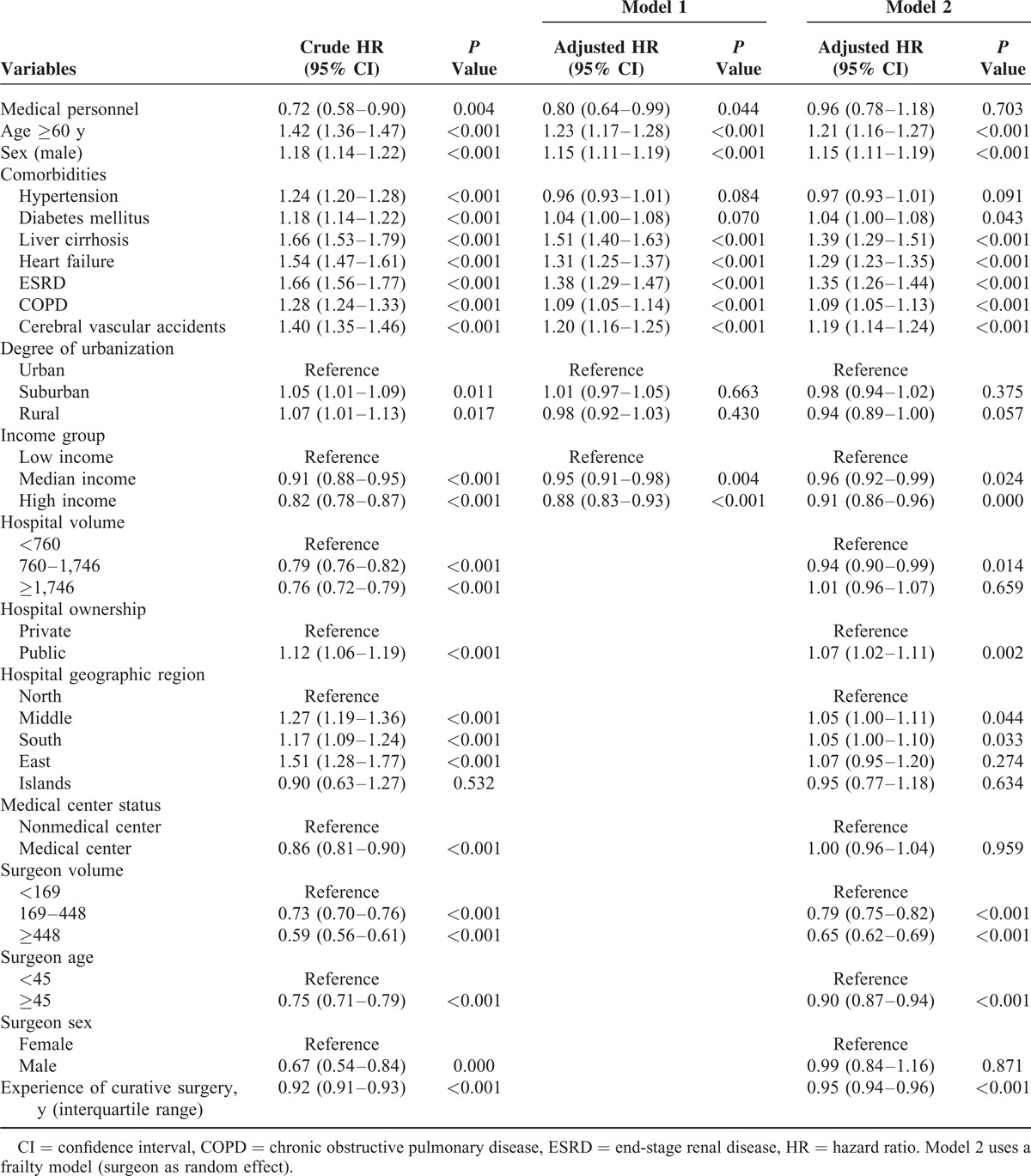
Risk Factors for 5-Year Mortality Between Medical Personnel and Nonmedical Personnel With Colorectal Cancer

## DISCUSSION

Our findings demonstrate that medical personnel patients have more favorable outcomes and lower resource utilization than nonmedical personnel patients. Furthermore, their long-term survival advantage may be attributable to their treatment providers. In this study, we demonstrated that the 5-year all-cause mortality was lower in medical personnel with CRC than in nonmedical personnel with the same disease, with a HR of 0.80 after adjustment of patients’ characteristics. The survival advantage narrowed partially after taking the characteristics of their treating physicians into consideration. Furthermore, medical personnel also had lower odds of hospital stay, ER visits within 30 days, and lower medical expenses, which could also be mitigated by adjusting for characteristics of hospitals and surgeons. These findings may provide good evidence supporting that medical personnel, the presumably better informed consumers, may have better outcomes through choosing better performing providers, including hospitals and surgeons.

To the best of our knowledge, this is the largest cohort study to discuss the association of resource utilization, outcome, and medical personnel in cancer surgery. The design of our study included an unbiased subject selection, strict diagnostic criteria, and linkage of several national registry data. Participation in NHI is obligatory, and all residents of Taiwan have access to healthcare with low copayments, so follow-up is comprehensive. The certification of CRC as a catastrophic illness requires histological confirmation. Because the medical expenses of patients with a certificate of catastrophic illness are reimbursed by NHI, the diagnoses, resource utilization, and outcome in our study were not only reliable but also exhaustive.

Unlike mandatory hierarchical referral systems in other countries, Taiwanese patients have the freedom to choose their preferred providers.^23,24^ Seeking second opinions and self-referrals after initial cancer diagnosis are common in Taiwan.^15^ Since no source of official performance data is available, patients tend to select surgeons or hospitals based on their own judgment and the recommendations of friends and relatives.^15^ Medical knowledge, familiarity with the healthcare system, and better patient–doctor communication of medical personnel may contribute to better outcomes.^25^ Medical personnel have more information about the reputations and outcomes of providers. Therefore, medical personnel might have an advantage of choosing better providers. Medical personnel are better informed savvy consumers^26,27^ and have ready access to high-quality care.^28^ Medical personnel may be treated differently from other patients because the health provider might feel pressure from these more informed patients; this also could be a sort of providing favors to colleagues.^29^

A growing body of studies has reported that high hospital and surgeon volume, especially surgeon volume,^30,31^ significantly reduces complications and improves cancer patients’ outcomes.^10,13,32^ Cornish et al^8^ reported that organizational structure impacted not only 30-day risk-adjusted mortality and length of stay but also at least 12 lymph nodes harvest and circumferential margin involvement, which were related to long-term outcome. Surgery volume–cost associations have been proven in different cancers.^23,33^ Lower costs potentially imply more efficient treatment or lower additional expenses for complications, which is correlated with shorter LOS.^34^ The information on hospitals and surgeons might not be fully revealed to the general population. Our study showed that medical personnel with CRC had higher odds of choosing high-volume hospitals and surgeons.

This study includes several limitations. First of all, several potential confounders, including obesity, tobacco use, alcohol use, and family history of malignancy, could not be found in the data. Second, we lacked data on tumor markers, histological features, genetic factors, and stage of cancers in these patients, which might affect patients’ long-term survival. Medical personnel might have higher accessibility to ambulatory care resources and have an increased chance of early diagnosis.^35^ However, they may easily overlook the importance of early detection and receive less cancer screening examinations^36^ and tend to make improper self-diagnoses with absolute certainty, especially in fields out of their own.^29^ The long-term survival in our study had no difference after adjusting for characteristics of patients and providers.

In conclusion, this nationwide population-based study shows that medical personnel receiving definitive surgery for CRC had better outcomes in comparison with the rest of CRC patients, which can be explained partially by the former choosing better hospitals and surgeons. By using definitive surgery for CRC as an example, the current findings may provide good evidence supporting that medical personnel have more information to help them choose better hospitals and surgeons. Outcome disparities between medical personnel and the rest exist. Policymakers should make a larger effort to minimize the information gap.
